# Injectable bioactive scaffold able to stimulate oral bone regeneration on demand

**DOI:** 10.1007/s10856-025-06879-2

**Published:** 2025-04-08

**Authors:** Anna Tampieri, Marta Tavoni, Teresa Vicidomini, Hina Inam, Elisa Restivo, Livia Visai, Umberto Romeo, Simone Sprio

**Affiliations:** 1 Institute of Science, Technology and Sustainability for Ceramics, Italian National Research Council (ISSMC-CNR), Faenza, Italy; 2 Department of Oral and Maxillofacial Sciences, Sapienza University of Rome, Rome, Italy; 3 Department of Chemistry, Life Sciences and Environmental Sustainability, University of Parma, Parma, Italy; 4 Molecular Medicine Department (DMM), Center for Health Technologies (CHT), UdR INSTM, University of Pavia, Pavia, Italy; 5 UOR6 Nanotechnology Laboratory, Department of Prevention and Rehabilitation in Occupational Medicine and Specialty Medicine, Istituti Clinici Scientifici Maugeri IRCCS, Pavia, Italy; 6 Interuniversity Center for the Promotion of the 3Rs Principles in Teaching and Research (Centro 3R), Operative Unit (OU) of University of Pavia, Pavia, Italy

## Abstract

Bone regeneration in oral surgery remains a challenge, due to the features of the oral environment, characterized by the presence of saliva and extensive interaction with external pathogens. Recent advances in this field highlighted that biomimetic apatites in which Ca^2+^ is replaced by Fe^2+^/Fe^3+^ ions are promising candidates to guide bone regeneration with on demand activation control. In this study the Fe-doped apatite nanoparticles (FeHA) were developed and compared with magnetite nanoparticles, as new magnetic bio-activator, to be embedded in apatitic injectable paste/cement. Upon self-hardening, the new injectable cement generates a mechanically competent 3D superparamagnetic scaffold, endowed with remote activation by using static magnetic fields. We investigated the alkaline phosphatase expression and activity, as well as the behaviour of cells, when seeded onto the scaffold. The results show the ability of the cement to stimulate cell colonization and differentiation and how, when magnetized, they can further boost such phenomena. The proposed devices, in association with a magnetic aligner, can represent a new approach in oral surgery, able to tune the bone remodelling on demand, when the regenerative potential is impaired by physiological conditions such as aging or chronic diseases.

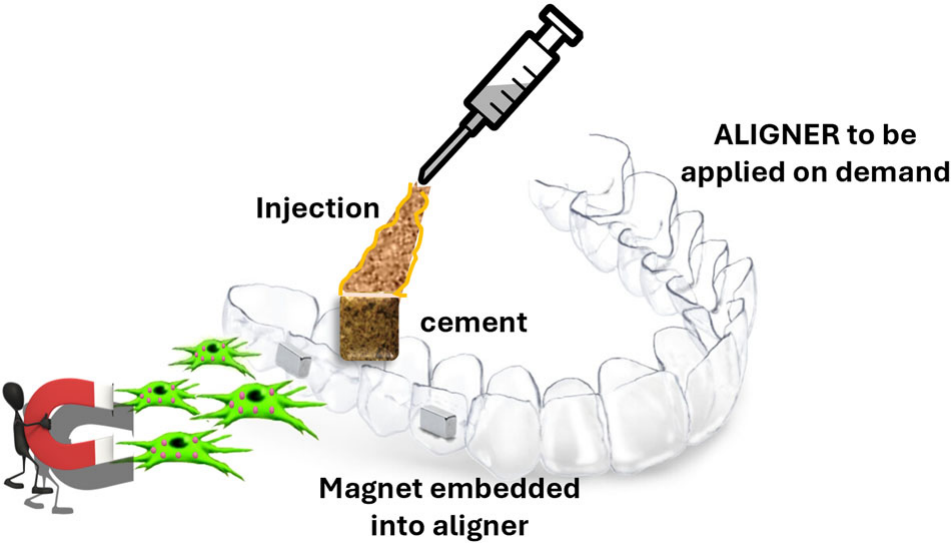

## Introduction

Bone regeneration is an important issue in medicine, especially considering the progressive aging of the population and the increasing need for such procedures [1]. Particularly referring to the dentistry field, a major problem is the insufficient amount of alveolar bone tissue needed for various surgical approaches including the application of dental implants or the treatment of peri-implant defects. This is also a result of periodontitis, which has become a prevalent disease that damages the supporting tissues of a tooth [2]. This highlights the need for regenerative strategies capable of effectively overcoming these challenges, considering that supra-alveolar bone regeneration is the most complex, due to limited resources in terms of vascularization and underlying tissues. Even more critical is the treatment of peri-implant defects with surrounding bone loss and peri-implantitis, which is responsible for implant failure. Similar issues are encountered in oral and maxillofacial surgery, particularly in the management of medication-related osteonecrosis of the jaw, an adverse reaction that causes progressive bone destruction with overlying mucosal ulceration [3]. Several protocols have been developed to restore bone loss: the most traditional approach relies on autologous grafts, which involve harvesting bone tissue from another part of the patient’s body. Although effective, this technique has limitations such as the limited availability of donor sites, prolonged surgical time, patient morbidity, quality of grafted bone, and risk of infections with bone resorption at the donor site, all factors contributing to variations in outcomes and treatment efficacy. For the upper jaw, another technique is sinus augmentation, also known as sinus lift or floor elevation, which aims to increase bone height and volume, often necessary to ensure the stability and longevity of the implant. However, this procedure is not free from complications, such as perforation of the Schneiderian membrane, infections, sinusitis, and variable results in terms of regenerated bone volume [4].

Nowadays, Guided Bone Regeneration (GBR) is widely applied and also used to restore alveolar bone [5]. The procedure involves the use of barrier membranes, either resorbable or non-resorbable, to isolate the area and prevent the infiltration of soft tissues while promoting the growth of new bone tissue using a supporting scaffold. Despite its widespread use in dentistry, factors such as the patient’s age, health status, low biocompatibility, infections, the anatomical position of the defect, and the surgeon’s expertise can influence the success of GBR, thus leading to unpredictable and variable results.

The need for new and simple surgical strategies in oral interventions to promote and sustain the bone regenerative process is more and more evident. Particularly, the use of synthetic grafts can make surgical procedures more accessible, predictable, and successful. Key elements to consider in synthetic biomaterials for oral applications include bioactivity, mouldability or minimally invasive injectability, high mechanical stability and ability to minimize immune responses, rejection or infections at the healing site.

Among synthetic bone grafts, apatite-based materials have gained popularity for their ability to provide a stable and osteoconductive framework for bone formation [6, 7]. Particularly when obtained as non-stoichiometric and in nanocrystalline form, apatites exhibit excellent biocompatibility and osteogenic ability, also showing antimicrobial ability [8, 9]. Of particular interest for the dental field is the achievement of malleable constructs that can fill complex-shaped alveolar or peri-implant bone defects. In this respect, apatitic bone cements are currently being investigated for their bioactivity, malleability, and self-hardening ability at physiological conditions.

Another aspect of ever-increasing importance is the treatment of patients with insufficient endogenous potential [10], including the elderly as well as patients affected by degenerative diseases such as periodontitis, all conditions where new bone formation is impaired and may significantly retard or nullify the bone regeneration. To this purpose, increasing attention was dedicated to magnetic biomaterials, able to be remotely activated on demand by using magnetic signals boosting the endogenous cell activity [11, 12]. The use of magnetic cement, particularly in the field of dentistry, is still largely unexplored and can represent an outstanding solution to significantly improve the clinical outcome and achieve effective and faster oral bone regeneration in a much wider patient population. In this respect, a recently developed apatitic phase (FeHA), endowed with intrinsic superparamagnetic properties given by the partial replacement of Ca^2+^ with Fe^2+^/Fe^3+^ ions has demonstrated excellent biocompatibility, osteogenic, and osteointegrative properties [13].

With these premises, the present work describes novel magnetic apatitic bone paste/cements obtained by functionalizing calcium phosphate precursors with FeHA nanoparticles to achieve tuneable, 3D injectable scaffolds for application in alveolar bone defects and/or maxillary sinus augmentation by mini-invasive procedures. On this basis, various paste formulations with different content of FeHA nanoparticles were optimized in terms of injectability, cohesion, and magnetization in a dental-like environment to maximise the final magnetic properties under static magnetic field and compared with the same cement formulation added with magnetite nanoparticles. The new cements were evaluated by preliminary in vitro tests to assess cytocompatibility, cell morphology and the osteogenic differentiation process. The perspective is to use in vivo magnetic aligners able to generate magnetization on demand in proximity of the bone defect, to stimulate oral bone regeneration.

## Materials and Methods

### Preparation of calcium phosphate cements (CPC)

The inorganic powder precursor and liquid components of the CPC were prepared according to a previous work [14]. Briefly, a metastable CaP precursor made of the α-polymorph of tricalcium phosphate phase (i.e. α-Ca_3_(PO_4_)_2_: αTCP) was prepared by mixing calcium carbonate (CaCO_3_, Sigma Aldrich, St. Louis, MO, United States) and dicalcium phosphate dibasic anhydrous (CaHPO_4_, Sigma Aldrich), followed by thermal treatment at 1400 °C for 1 h and rapid cooling to prevent the re-crystallization of the β-polymorph of TCP (βTCP), as a non-reactive inorganic phase. The obtained powder was ground by planetary mono mill (Pulverisette 6 classic line, Fritsch, Germany) for 50 min at 400 rpm using a zirconia jar with 2 mm diameter grinding media. The liquid component of the paste (setting solution) was made of aqueous solution of disodium hydrogen phosphate dihydrate (Na_2_HPO_4_ ∙ 2H_2_O, Fluka) and sodium alginate (Alginic Acid Sodium Salt from Brown Algae, Sigma Aldrich).

### Synthesis of superparamagnetic FeHA powder

FeHA powder was prepared following a previously reported procedure [15]. Briefly, a phosphoric acid (Aldrich, 85 wt.% pure, 44.40 g in 300 mL H_2_O) solution was added dropwise into a basic suspension of calcium hydroxide Ca(OH)_2_ (Aldrich, 95 wt.% pure, 50 g in 400 mL H_2_O) containing Fe ions, over a period of 2 h, under constant heating and stirring. FeCl_2_ ∙ 4H_2_O (Aldrich, 99 wt.% pure, 12.74 g in 75 mL H_2_O) and FeCl_3_ ∙ 6H_2_O (Aldrich, 97 wt.% pure, 17.86 g in 75 mL H_2_O) were added together as sources of Fe^2+^ and Fe^3+^ ions during the neutralization process. The total amount of Fe ions with respect to Ca ions was adjusted to obtain Fe/Ca = 20 mol.%. The reaction temperature was kept below 40 °C to prevent the crystallization of iron oxides. The reaction products were kept in suspension by constant stirring and heating for 1 h, then left to age for 24 h at room temperature without further stirring. The precipitate was separated from mthe other liquor by centrifugation, then washed with distilled water and centrifuged three times; finally, it was freeze-dried and sieved at 150 microns, ready for mixing with the αTCP precursor.

### Preparation of the superparamagnetic cements

The magnetic apatitic cement (MagCPC) was prepared by dry mixing the FeHA powder with the αTCP precursor in amounts relevant to elicit superparamagnetic properties in the final construct. Various formulations of the cement were obtained by using different content of FeHA, alginate and phosphate concentration in the setting solution, by mixing appropriate amounts of powder and liquid (i.e. establishing defined liquid to powder ratio (LP)) to obtain a paste able of self-hardening in artificial saliva within a timelapse suitable for appropriate management in the clinical scenario.

MagOxCPC was prepared by adding magnetite nanoparticles (Aldrich, Iron (II, III) oxide, powder, <5 micron, 98%) to the solid precursors of CPC in an amount suitable to introduce the same molar amount of iron as MagCPC.

### Physicochemical characterization

The phase composition of the MagCPC precursors and final cements was obtained by X-ray diffraction (XRD) using a D8 Advance diffractometer (Bruker, Karlsruhe, Germany), with CuKα radiation, 2θ range 10–80, and scan step 0.02. The artificial saliva was prepared as a modified Tani-Zucchi solution containing potassium chloride (KCl) at 20 mM, potassium thiocyanate (KSCN) 5.3 mM, sodium phosphate (Na_2_HPO_4_) 1.4 mM, sodium bicarbonate (NaHCO_3_) 15 mM, and lactic acid 10 mM [16–18]. MagCPC was immersed in the artificial saliva at 37 °C under shaking conditions. At scheduled times (2 h, 1 day, 3 days, and 7 days), one sample for each formulation was collected and immersed in ethanol to halt the phase transformation, freeze, and analysed by X-ray diffraction to evaluate the phase evolution of the αTCP precursor into the hydroxyapatite phase.

Quantitative chemical analysis was made using inductively coupled plasma–optical emission spectrometry (ICP-OES Agilent Technologies, Santa Clara, CA, USA), to determine the overall content of Ca, P and Fe in the cements as well as the extent of released ions. Samples were previously prepared as follows: 20 mg of powder were dissolved in 2 mL of HNO_3_ (Aldrich, 65 wt.% pure) and the solution volume was increased up to 100 mL with deionized water. The obtained values were expressed in terms of Ca/P, (Ca+Fe)/P, and Fe/(Ca+Fe) mol%, as well as the total Fe wt%. Concerning the ions release, bone cements were incubated with 0.01 M HEPES solution (pH 7.4) for up to 7 days. At the scheduled time point, 100% of the supernatant was collected, analysed and refreshed with the same volume of fluid.

To determine the content of Fe^2+^ in the FeHA, 10 mg of the FeHA powder was dissolved in 1 mL of HCl 0.5 M and added to a 100 mL volumetric flask, together with 5 mL of 1, 10-phenanthroline. Subsequently, 10 mL of citrate buffer (0,1 M, pH: 4) was added, and the resulting solution was kept for 5 min under sonication for full colour development. The solution was diluted each to 100 mL with distilled water. UV-Vis absorption spectra were recorded in the range of 400 – 700 nm (Abs. maximum at 510 nm ca) and the Fe^2+^ concentration was calculated using a previously performed calibration [19]. The content of Fe^3+^ was obtained by the difference between the overall Fe content obtained by ICP-OES and the Fe^2+^ content.

Fourier-transform infrared spectroscopy with attenuated total reflection (FTIR-ATR) (Nicolet iS5, Thermo Scientific) was investigated in the range 400–4000 cm^−1^.

Dynamic light scattering (DLS) (Malvern, Zetasizer Nano ZSP) was performed to investigate the zeta potential of the samples by dispersing NPs (0.2 mg/mL) in citrate solution (67 mM) at pH 7–8.

The initial and final setting times of the cement formulations were monitored by Gillmore needles, according to standard ASTM C266-99.

The morphology of MagCPC was explored by Scanning Electron Microscopy (SEM), using a Zeiss EVOMA10 scanning electron microscope (Carl Zeiss, Oberkochen, Germany) at 20 kV acceleration voltage.

The porosity percentage of the cements was evaluated on hardened cylindrical specimens as *P* = 1 - ρ/ρ_0_, where ρ is the density of the specific cement, calculated as weight-on-volume ratio and ρ_0_ is the maximum density of the specific cement (i.e. in case of no porosity), in turn evaluated by the formula:

$${{\rho}}_{{0}}\,{{=}}\,{{\sum}}{{{\rho}}_{{i}}}{{{X}}_{{i}}}$$

where ρ_i_ is the theoretical density of the *i*th crystalline phase composing the cement and x_i_ is the weight fraction of the i^th^ crystalline phase in the specific cement, as obtained by XRD analysis.

The evidence of FeHA magnetization was evaluated with Nd-Fe-B magnet with a field of 1.4 T. Hysteresis cycles of FeHA were quantified utilising AGFM (Alternating Gradient Force Magnetometry) at room temperature. Ultra-thin plastic substrates of approximately 3 ×3 mm² in size were utilised. FeHA nanoparticles (0.10–0.30 mg) was fixed to the substrate with a minimal amount of adhesive. This technique enabled the measurement of the magnetisation curve of FeHA powder in hysteresis cycle mode (from +H_max_ to -H_max_ and back) in a magnetic field H_max_ of 1 T. Magnetization of bone cements at lower field was measured at 3.4·10−^3 ^N·A−^1^m−^1^ via YSZ 01C/02C Susceptometer (Sartorius Mechatronics, Italy). Testing was conducted on cylindrical samples with a diameter of 11 mm, a height of 2 mm, and an average weight of 300 mg. Each sample was analysed in a class E1, corresponding to a magnetic field of 2700 A/m and a distance from the magnet of 18 mm, at both the S and N position of the magnet.

The compressive strength of the CPC, MagCPC20 and MagOxCPC cements was evaluated by testing cylindrical specimens (n. 5 samples for each type; diameter = 8 mm; height = 17 mm) obtained after hardening in teflon moulds for 30 min. The tests were performed in displacement control at 1 mm/min by a universal testing machine (MTS Insight 5, Eden Prairie, MN, USA).

### Biological analyses

#### Cell culture conditions

Human osteosarcoma SaOs-2 osteoblast-like cell line (HTB-85) was obtained from the American Type Culture Collection (ATCC, USA). Cells were cultured in proliferative medium based on McCoy’s 5 A modified medium supplemented with 15% foetal bovine serum, 1% L-glutamine, 0.4% penicillin/streptomycin, 0.2% amphotericin B, 2% sodium pyruvate [20]. Cells were cultured at 37 °C, with 5% CO_2_ and routinely trypsinized once they reached confluency. They were counted and seeded onto sterile CPC, MagCPC20 and MagOxCPC hardened cements.

#### Cell viability

SaOs-2 cells (100,000 cells/sample) were seeded on each cement either in the absence or presence of a static magnetic field (SMF) of 320–350 mT, applied by ferritic magnets placed under the culture plates. They were incubated with proliferative medium for 24 h, 72 h and 168 h at 37 °C, with 5% CO_2_. After each incubation time, the pH value of cell medium was measured and remained physiological. The samples were gently washed with sterile phosphate-buffered saline (PBS) 1× (0.134 M NaCl, 20 mM Na_2_HPO_4_, 20 mM NaH_2_PO_4_, pH 7.4), transferred to clean wells and the viability was evaluated through Cell Counting Kit-8 (CCK-8) colorimetric assay. Briefly, 10% of the CCK-8 solution was diluted into McCoy’s 5 A base medium and incubated on the samples for 4 h at 37 °C (+5% CO_2_). Aliquots of 100 µL were read with a CLARIOstar plate reader (BMG Labtech, Germany) at 460 nm wavelength. Titration curve interpolation was used to express the number of cells on each sample [21]. Data were represented as a percentage of cell viability on MagCPC20 and MagOxCPC compared to CPC. The experiment was performed in triplicates. A one-way analysis of variance (ANOVA), followed by Bonferroni’s multiple comparisons test within samples, was performed. *p* values < 0.05 were considered statistically significant.

#### Protein extraction and Enzyme-linked immunosorbent assay (ELISA)

SaOs-2 (100,000 cells/sample) were seeded and cultured for 24 h, 72 h and 168 h in proliferative medium, as previously described. After 168 h, the cements were gently washed with PBS 1×, transferred into clean wells and incubated on ice for 20 min with 500 μL of a solution containing a radio-immunoprecipitation (RIPA) assay 1× lysis buffer, 1 mM sodium orthovanadate, and 1 mM protease inhibitor. The lysis solution, containing the proteins, was sonicated for 5 min at room temperature (RT) and centrifuged for 10 min at 15,000 rpm, at 4 °C. The total protein concentration was evaluated with a bicinchoninic acid (BCA) protein assay kit according to the manufacturer’s instructions. To measure the amount of alkaline phosphatase (ALP), an enzyme-linked immunosorbent assay (ELISA) was performed on the extracted proteins. Briefly, ELISA microtiter wells were coated, overnight at 4 °C with 100 µL of extracted proteins (10 µg/mL in coating buffer). After three washes with PBST (PBS 1× + 0.05% (v/v) Tween 20), the wells were blocked with 3% (w/v) bovine serum albumin (BSA) for 1 h at RT. BSA-coated wells were used as negative controls. The wells were subsequently incubated for 1 h at RT with 100 µL with anti-ALP polyclonal rabbit antisera (1:1000 dilution in 1% BSA) [22]. After washing, 100 µL of horseradish peroxidase (HRP)-conjugated goat anti-rabbit IgG (1:1000 dilution in 1% BSA) were incubated at RT for 1 h. After washing, the reaction was developed with o-Phenylenediamine dihydrochloride (OPD) tablets dissolved in double distilled water. The absorbance was read at 450 nm with a reference wavelength of 620 nm at CLARIOstar microplate reader. The obtained values of each sample were plotted with a calibration curve containing known amounts of protein. The amount of ALP protein expressed by cells adherent on the cements was reported as micrograms per cement ± standard deviation (SD). A one-way analysis of variance (ANOVA), followed by Bonferroni’s multiple comparisons test between samples, was performed.

#### Alkaline Phosphatase (ALP) activity assay

ALP activity assay was performed with SaOs-2 cells on day 7 of culture in proliferative medium. ALP content was determined using a colorimetric assay as described by Riva et al. [23]. In brief, after 7 days of culture, samples were gently washed with PBS 1 × and transferred into clean wells. 500 µL of 0.3 M p-nitrophenyl phosphate (pNPP) (dissolved in glycine buffer, 0.1 M, pH 10.5) were added to each sample, including cements without cells used as negative controls. After 30 min of incubation at 37 °C, the reaction was stopped by the addition of 50 µL 5 M NaOH. Aliquots of 100 µL were read at 415 nm wavelength and the absorbance was compared with a calibration curve of p-nitrophenol standards. The enzyme activity was expressed as micromoles of p-nitrophenol produced per minute per microgram of ALP protein and was reported as a percentage of ALP activity on MagCPC20 and MagOxCPC compared to CPC ± SD. The experiment was performed in triplicate and repeated twice. A one-way analysis of variance (ANOVA), followed by Bonferroni’s multiple comparisons test between samples, was performed. *p* values < 0.05 were considered statistically significant.

### Microscopic analysis of the cells

#### Confocal laser scanning microscopy (CLSM) analyses

Cells were seeded as previously described. After 24 h of culture in proliferative medium, the scaffolds were gently washed with PBS 1× and cells were fixed with 4% (w/v) paraformaldehyde solution for 20 min at RT and washed with PBS 1× three times. Cells were permeabilized with triton 0.1% (v/v) for 5 min at RT, washed with PBS 1× and then incubated with 3% BSA for 1 h at RT. The samples were incubated with Alexa Fluor 488-conjugated primary mouse anti-β-tubulin antibody (diluted 1:20 in BSA 1%) for 1 h at RT. At the end of the incubation and the following washes, cellular nuclei were counterstained with a solution of Hoechst 33342 (2 μg/mL) for 20 min at RT and then washed with PBS 1×. Cells attached on cements were observed using CLSM (Leica, model TCS SP8 DLS, Leica, Germany) using a 40 × oil immersion objective. Images by using a 2.5 electron zoom were acquired. Orthogonal projections were obtained by using the software LAS X (Leica Microsystems, Wetzlar, Germany).

#### Scanning electron microscopy (SEM) analyses

Cells were seeded as previously described. After 7 days of culture in proliferative medium, the scaffolds were gently washed with PBS 1× and cells were fixed with 2.5% glutaraldehyde (v/v in 0.1 M sodium cacodylate, pH 7.2) for 1 h at 4 °C. After two washes in sodium cacodylate for 10 min, the samples were dehydrated with ethanol (25%, 50%, 75% and 96%). Each wash was performed for 3 min. The samples were lyophilized for 3 h using a K-850 apparatus (Emitech Ltd., Ashford, UK) and placed on a mounting base. Finally, they were gold sputtered, and images were acquired using a Zeiss EVO-MA10 scanning electron microscope (Carl Zeiss, Oberkochen, Germany), 20 kV acceleration voltage.

## Results

### Characterization of the Fe^2+^/Fe^3+^-doped hydroxyapatite nanoparticles (FeHA)

The chemical analysis of FeHA nanoparticles by ICP (Table 1) confirms the presence of Fe ions substituting Ca^2+^ in amounts very close to the one nominally introduced in the reaction vessel (i.e., ≈97%). The molar Ca/P ratio in FeHA, lower than the theoretical value of HA (i.e. Ca/P = 1.67), reports on calcium decreasing due to Fe ions substitution, whereas the higher (Ca+Fe)/P ratio indicates that besides Ca^2+^ and Fe ions in substitutional positions, also Fe ions in interstitial positions are present.

**Table 1:** ICP-OES, UV-Vis and DLS analysis of FeHA nanoparticles

**ICP-OES**	**Ca/P**	1.47 ± 0.01
	**(Ca** + **Fe)/P**	1.78 ± 0.02
	**Fe/(Ca** + **Fe) mol%**	17.47 ± 0.59
	**Fe tot wt.%**	13.02 ± 1.75
**UV-Vis**	**Fe**^**2+**^ **% (Fe**^**2+**^**/Fe tot)**	5.76
	**Fe**^**3+**^ **% (Fe**^**3+**^**/Fe tot)**	94.24
**DLS**	**ζ-potential (mV)**	−29.20 ± 0.50
	**Z-average (nm)**	201 ± 1
	**PdI**	0.37 ± 0.02

**Fig. 1:**
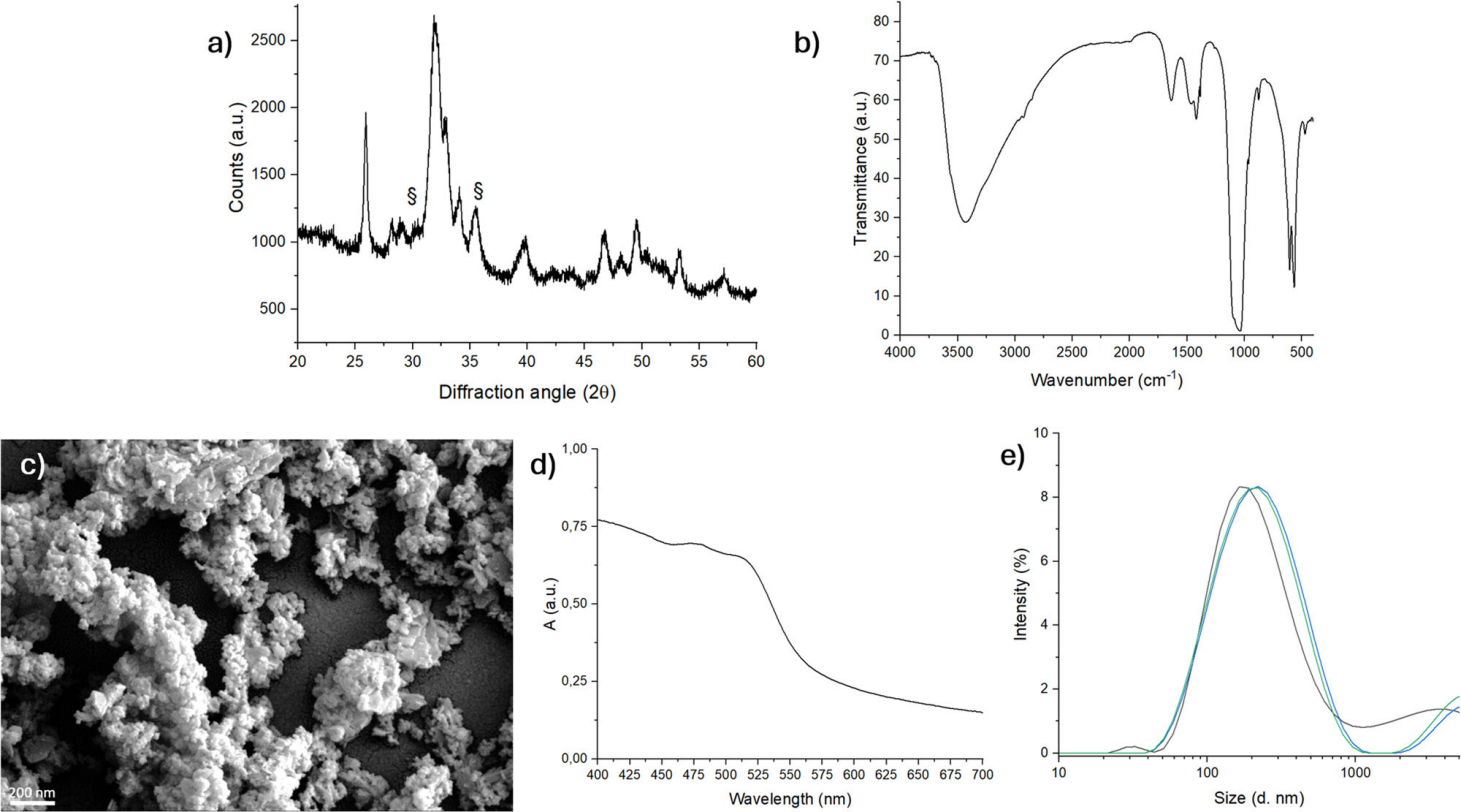
Physico-chemical and morphological analysis of FeHA nanoparticles. (**a**) XRD pattern (§ indicates the XRD peaks of magnetite phase), (**b**) FTIR analysis, (**c**) SEM image (scale bar 200 nm); (**d**) UV-Vis spectra, indicating the presence of Fe^2+^; (**e**) FeHA particles size

Figure 1a exhibits the typical X-ray diffraction pattern of hydroxyapatite phase (ICDD file no. 09–0432). The peak broadening can be ascribed to the presence of very small crystalline domains, as typical of ion-doped apatites synthesized at low temperature. Additionally, a low amount of magnetite phase was detected (ICDD file no. 19–0629), estimated as ∼9 wt% by semiquantitative XRD analysis.

The FTIR spectrum of FeHA (Fig. 1b) exhibits the typical profile and absorption bands of apatite, particularly evidencing the presence of ν_1_, ν_2_, ν_3_ and ν_4_ stretching modes of phosphate groups forming the hexagonal apatite lattice at 963, 472, 1040 and 560–600 cm^−1^, respectively, as well as shoulders reporting to PO_4_^3−^ and HPO_4_^2−^ ions present in a non-apatitic environment, a typical feature in nanocrystalline apatites [19]. The presence of carbonate ions in the samples was confirmed by the presence of a very weak carbonate band at ca. 870 cm^−1^ and by two weak bands at 1415 and 1455 cm−^1^, suggesting a B-type carbonate doping (CO_3_^2−^ ions substituting PO_4_^3-^ ions). B-carbonation was expected because carbonate ions enter the crystal lattice substituting phosphate when apatites are prepared by wet precipitation [24]. Moreover, the broad IR absorption bands with a splitting factor = 3.07 confirm the low crystal ordering of the as-obtained FeHA, similar as previously reported for various ion-doped nanocrystalline apatites [19]. The SEM analysis of the FeHA powder (Fig. 1c) shows primary particles with size of 5–20 nm, thus confirming the small crystal domain size of FeHA. SEM images also show that the primary particles are agglomerated in clusters of about 5–10 μm, due to electrostatic interaction between the FeHA nanoparticles, as typical of nanosized, nanocrystalline apatites. The amount of Fe^2+^ in FeHA was assessed by means of colorimetric method (Fig. 1d) by reading the absorbance at 510 nm and calculating the Fe^2+^ concentration on the basis of the previously obtained calibration curve, whereas by difference from the overall content of Fe, obtained by ICP-OES, permitted to obtain the content of Fe^3+^.

Despite the tendency to aggregate, FeHA shows good dispersibility in fluid media, attested by the quite low ζ potential (−29.20 ± 0.50) (Table 1) and the small particle size (Z-average = 201 ± 1 nm) (Fig. 1e).

**Fig. 2:**
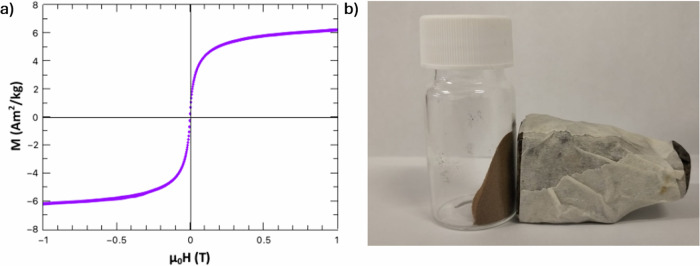
Magnetic evaluation of FeHA nanoparticles. (**a**) magnetic curves in function of the applied magnetic field; (**b**) Evidence of FeHA magnetization with a field of 1.4 T emitted by a Nd-Fe-B magnet

Figure 2a shows the magnetization curve for FeHA, which exhibits superparamagnetic-like behaviour, with magnetization of saturation of ∼6 Am^2^/Kg, while Fig. 2b shows evidence of FeHA powder attracted by a Nd-Fe-B magnet.

### Characterization of the bioactive MagCPC paste/cement

**Fig. 3:**
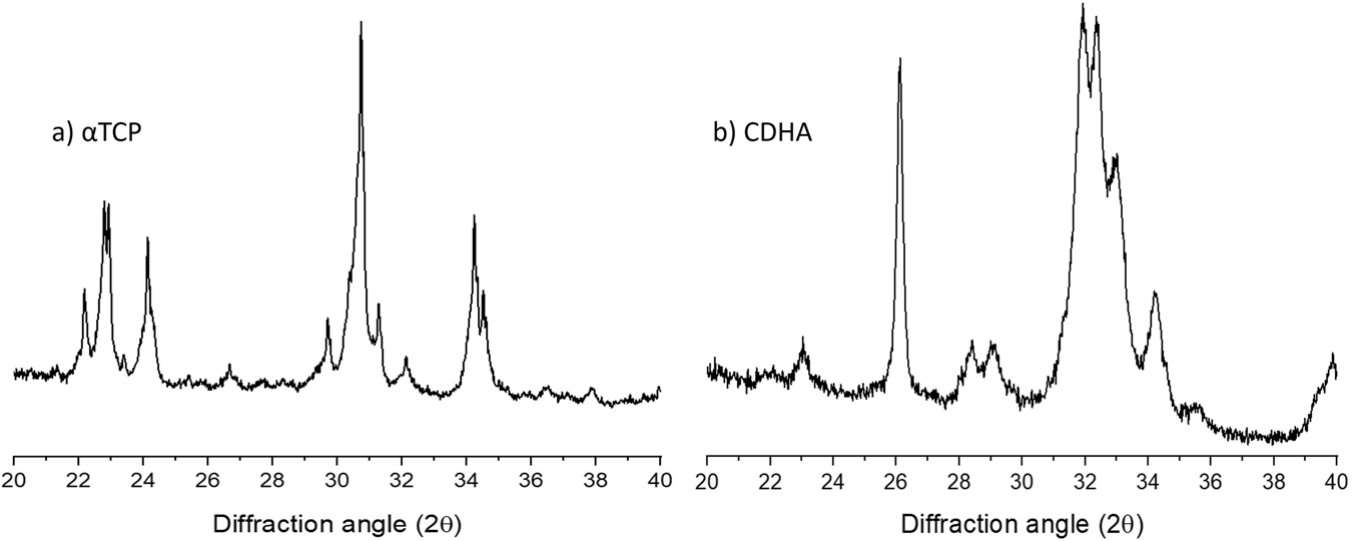
X-ray diffraction pattern of the (**a**) αTCP cement precursor and (**b**) set bone cement

The MagCPC was obtained by a dissolution-reprecipitation process occurring when a mixture of αTCP precursor with different amounts of FeHA powder was put in contact with an aqueous medium. The process gives rise to transformation of the αTCP precursor (Fig. 3a) in calcium-deficient hydroxyapatite (CDHA) (Fig. 3b), typically characterized by elongated nanocrystals that physically interlock giving rise to the hardening of the cement construct (setting) [14].

### Setting times, injectability and cohesion in fluids

The usability of regenerative bone cements is related to the optimization of some inter-related parameters such as the injectability (the ability to flow throughout a cannula/syringe), cohesion (the ability to not de-mix into solid and liquid components upon injection), hardening time (setting), porosity and mechanical properties [25]. In addition, any modification of the composition of the solid precursor affects its chemical reactivity and thus, its hydraulic behaviour responsible of the cement setting. In this respect, a series of experiment was conducted to assess the maximum amount of FeHA nanoparticles that could be homogenized with the αTCP precursor without significant alterations in setting times and injectability (an initial setting time of approximately 15–20 min is considered as suitable to meet the clinical practice requirements) [25–27]. In addition, we have optimized the amount of FeHA added to αTCP precursor to obtain MagCPC cement endowed with appreciable magnetization under a magnetic field in the range 0.3 to 1 T. The final cements were obtained by keeping the liquid-on-powder ratio to 0.75, the amount of alginate to 2 wt.% and the phosphate concentration in the setting solution to 2.5 wt.%, to yield a MagCPC cement able of self-hardening in artificial saliva within ∼20 min.

**Fig. 4:**
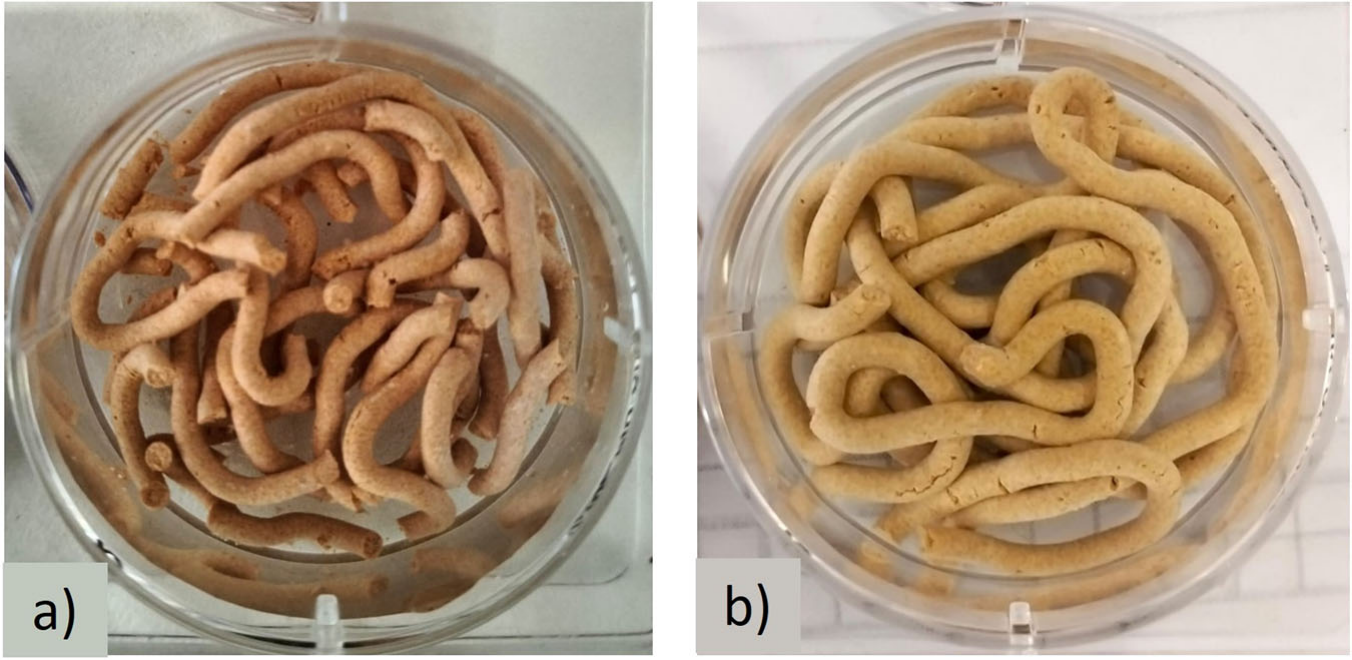
Cohesion of the magnetic cement containing 20% of FeHA and aqueous setting solution made of (**a**) pure water or (**b**) enriched with 2 wt% of sodium alginate after injection in artificial saliva

The incorporation of FeHA nanoparticles into the cement formulation resulted in a small reduction in both the initial and final setting times. A higher reduction of setting times was observed with the sample containing 30 wt% of FeHA nanoparticles. Any greater concentration of FeHA nanoparticles yielded inadequate paste workability and injectability (as well as setting times). For further characterization tests, we selected the cement containing 20% of FeHA for the preparation of the MagCPC. Moreover, we observed that the presence of alginate in MagCPC increased the paste cohesion (see Fig. 4b), without de-mixing or fragmentation after 30 min of immersion into artificial saliva at 37 °C. The compression strength of the hardened cement in dry conditions was MagCPC20 = 35 ± 2 MPa, to be compared with the reference CPC = 30 ± 3 MPa. For the sake of comparison, MagOxCPC was prepared in a similar manner, adding 20 wt% of magnetite, resulting in a compressive strength of 23 ± 3 MPa.

The magnetic properties of the final bone cements were quantified and reported in Table 2; the magnetisation value of MagCPC20 and MagOxCPC did not significantly change after aging in physiological fluid at 37 °C for 2 months. As expected, the iron-free cement (CPC) exhibited no magnetisation.

**Table 2:** Relationship between FeHA content, setting times and magnetization in magnetic samples

Sample	FeHA content (wt%)	Initial setting time (minutes)	Final setting time (minutes)	M (emu/g)
**CPC**	0	22 ± 2	93 ± 3	0.00
**MagCPC5**	5	16 ± 1	78 ± 3	0.04
**MagCPC10**	10	17 ± 2	77 ± 2	0.08
**MagCPC20**	20	18 ± 1	82 ± 2	0.13
**MagCPC30**	30	10 ± 1	32 ± 2	/
**MagOxCPC**	Magnetite = 20	27 ± 3	50 ± 2	0.55

**Fig. 5:**
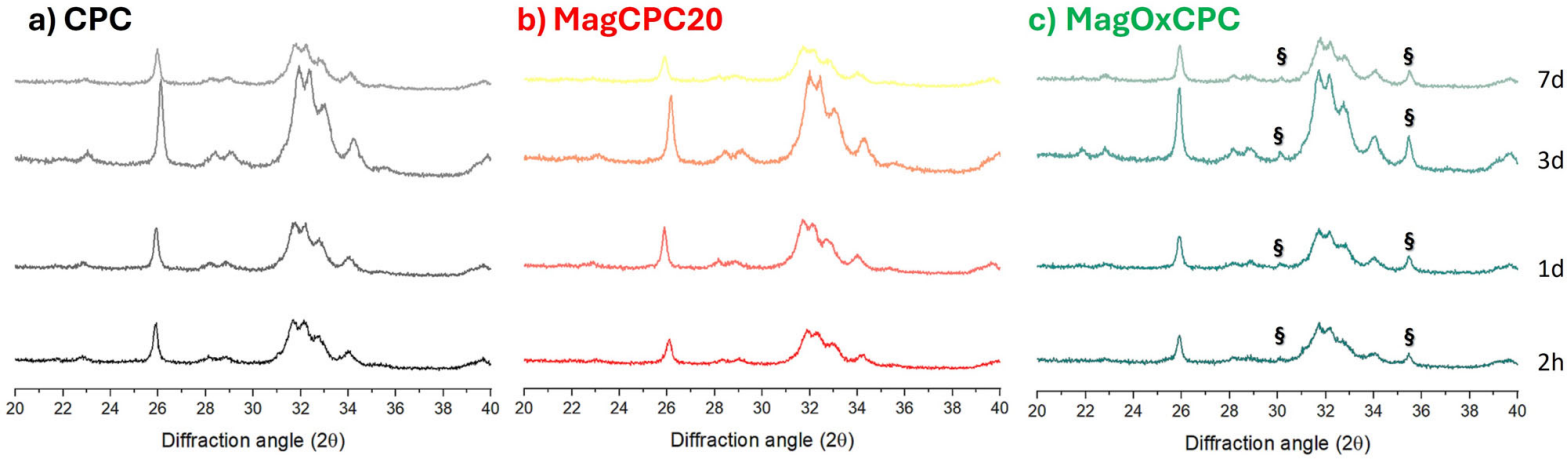
Evolution of the phase composition in the new cements. (**a**) control bone cement (CPC, black line), (**b**) magnetic bone cement containing 20% of FeHA nanoparticles (MagCPC20, red line) and (**c**) magnetic bone cement containing magnetite nanoparticles (MagOxCPC, green line, magnetite secondary-phase is marked with §) after incubation in artificial saliva at 37 °C up to 7 days

The setting process is accompanied by the transformation of the precursor into calcium-deficient HA. In this respect, the phase transformation accompanying the MagCPC20 cement hardening was monitored by XRD analysis, keeping the cement under incubation into artificial saliva at 37 °C in a thermostatic water bath up to 7 days, and monitoring the transformation of the αTCP precursor into HA phase at defined time-points. The amount of αTCP, hydroxyapatite and iron oxide secondary phases was quantified by JCPDS file 029–0359, 009–0432 and 19–0629, respectively. As illustrated in Fig. 5, the incorporation of FeHA nanoparticles did not disturb the dissolution/reprecipitation process by which the precursors transform into hardened apatitic cements, so the kinetics of the transformation process was not affected and completely occurred within 24 h. As the final MagCPC20 cement did not show any secondary phases, it can be devised that the Fe^2+^/Fe^3+^ remain as doping ions in the apatite crystals after the cement setting. In the case of MagOxCPC, the initial content of added magnetite remained unchanged.

Table 3 lists the cement composition in terms of Ca and Fe wt.% total amount as obtained by ICP-OES analysis. The analysis confirms that all the cements have comparable content in calcium, in particular MagOxCPC has a slightly reduced Ca content as it contains added iron oxide nanoparticles.

**Table 3:** Ca and Fe wt% total amount in CPC, MagCPC20 and MagOxCPC

	Ca wt%	Fe wt%
CPC	34.6 ± 2.0	–
MagCPC20	33.5 ± 2.0	1.1 ± 0.1
MagOxCPC	31.2 ± 1.0	2.0 ± 0.1

### Microstructural analysis

**Fig. 6:**
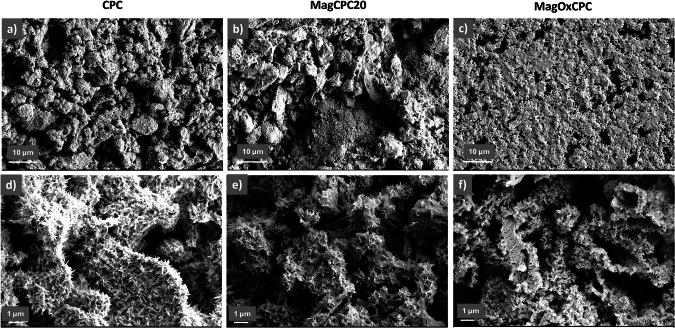
SEM micrograph of the hardened (**a**, **d**) CPC, (**b**, **e**) MagCPC20 and (**c**, **f**) MagOxCPC

SEM micrographs in Fig. 6 show the microstructure of CPC reference (Fig. 6a, d), MagCPC20 (Fig. 6b, e) and MagOxCPC (Fig. 6c, f), after setting and complete phase transformation. As an effect of the transformation process, the dissolution of the αTCP precursor particles, whose size was ∼1–2 μm, resulted in the formation of micropores in the final cement. The overall porosity extent of hardened cements was: CPC: 46.7% ± 2.6%; MagCPC20: 31.2% ± 1.8%; MagOxCPC: 57.9% ± 0.2%, the theoretical densities for hydroxyapatite and magnetite phase being 3.16 g·cm^−3^ and 5.21 g·cm^−3^, respectively.

### Ion release

**Fig. 7:**
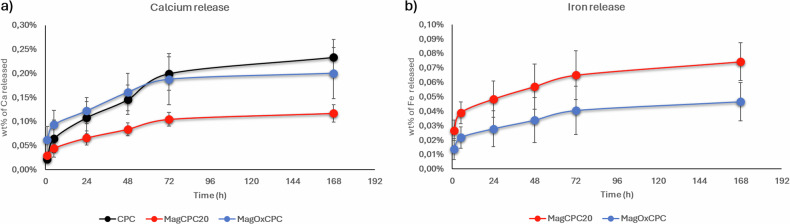
Calcium (**a**) and iron (**b**) ions wt% releases from CPC (black line), MagCPC20 (red line) and MagOxCPC (blue line)

The release of Ca and Fe ions, known to affect the bio-chemism of bone remodelling [28, 29], was evaluated by incubating bone cements in a 0.01 M HEPES buffer solution (pH 7.4) at 37 °C, afterwards the supernatants were analysed by ICP-OES. The data were expressed as the percentage of the weight of the cation released in relation to the total amount of the cation present in the cement formulation. Figure 7a shows that MagCPC20 releases less calcium than CPC and MagOxCPC. This difference could be ascribed to the presence of FeHA nanoparticles in MagCPC20 formulation, which increases the cohesion of the cement and reduces the overall porosity of the construct compared to CPC and MagOxCPC. Conversely, the release of Fe ions has an opposite trend where MagCPC20 releases more Fe ions than MagOxCPC (Fig. 7b). This difference could be associated to the much higher stability of magnetite contained in the MagOxCPC compared to the FeHA phase embedded in the MagCPC20.

### In vitro biological performance of magnetic cements

The human SaOs-2 cell line was selected due to its osteoblast-like properties, in contrast to other cell lines these cells can be grown rapidly in vitro [30] and are used to study proliferation and differentiation [31].

**Fig. 8:**
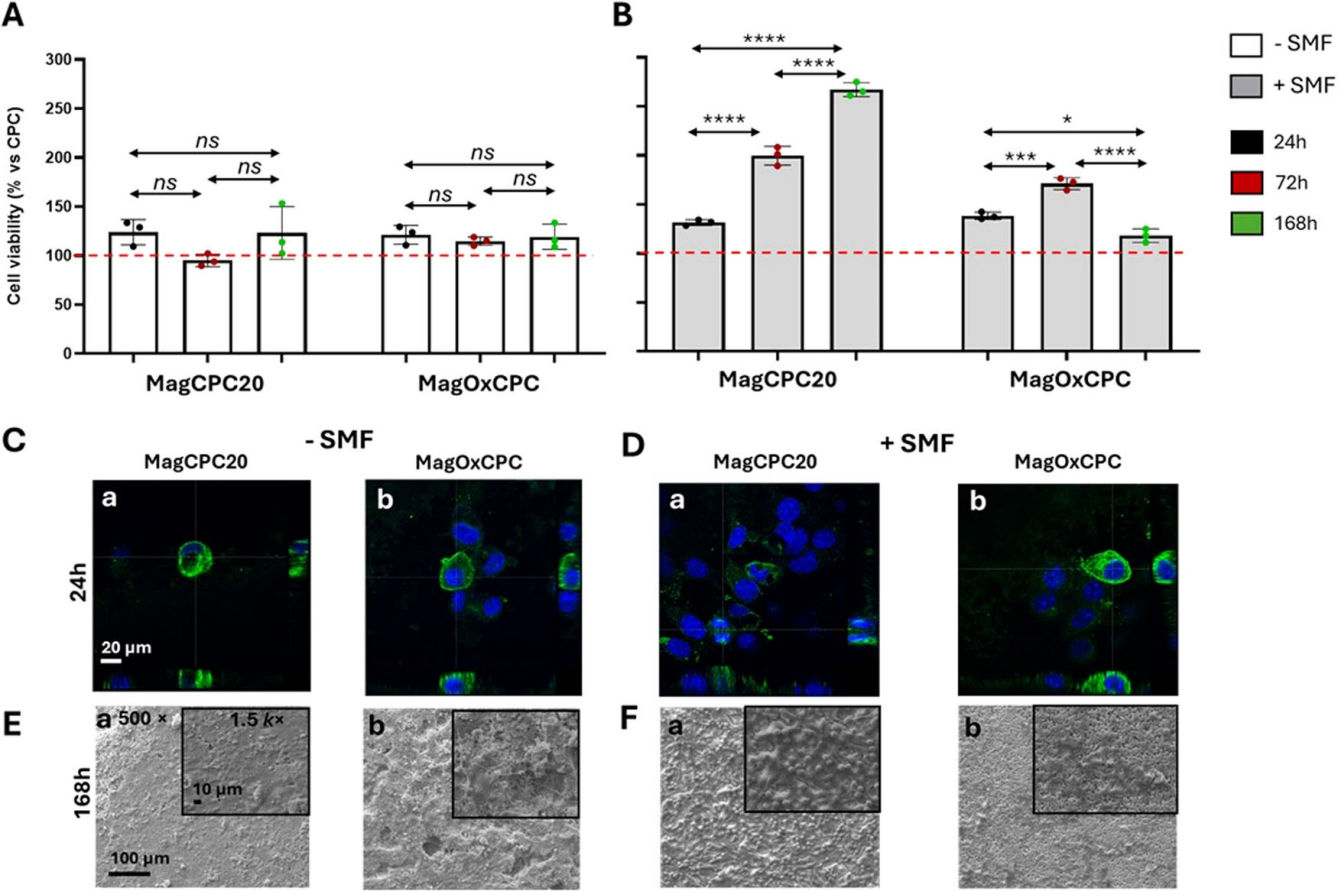
Cell viability, morphology and distribution. The viability data (**A**, **B**) were represented as the percentage of cell viability on MagCPC20 and MagOxCPC with respect to the CPC cement used as control (red dotted line, set as 100% of cell viability) in absence (**A**) and presence (**B**) of SMF. Bars indicate the mean values ± SD of results from three measurements in two separated experiments. Statistical analysis was performed using one-way analysis of variance (ANOVA), followed by Bonferroni’s multiple comparison test between samples. Statistically significant differences (*p* < 0.05) were observed: *p* < 0.05 (*), *p* < 0.001 (***) and *p* < 0.0001 (****). No significant differences (ns) were observed (*p* > 0.05) in SMF (-). Cell morphology (**C**, **D**) and distribution (**E**, **F**) images of cells on MagCPC20 (Ca; Da; Ea; Fa) and MagOxCPC (Cb; Db; Eb; Fb) were acquired through CLSM (**C**, **D**) after 24 h and through SEM (**E**, **F**) after 168 h of incubation in SMF (-) (**C**; **E**) and SMF (+) (**D**; **F**) conditions. **C**, **D** – Images acquired to evaluate cell adhesion after 24 h, with 2.5 electron zoom of 40 × magnification (scale bar 20 µm) on MagCPC20 (Ca; Da) and MagOxCPC (Cb; Db). **E**, **F** – SEM images of cells on MagCPC20 (Ea; Fa) and MagOxCPC (Eb; Fb) were acquired at 500 × magnification (scale bar 100 µm) and insets at 1.5k× magnification (scale bar 10 µm)

**Fig. 9:**
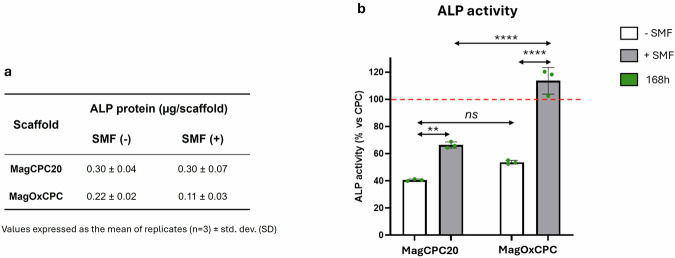
Alkaline phosphatase quantification (**a**) and activity (**b**) after 7 days of cell incubation on cements. a): the proteins were extracted, and the quantified alkaline phosphatase (ALP) was reported as micrograms produced on cements. Data were expressed as the mean values ± SD of results from three measurements in two separate experiments. No statistically significant differences were observed between samples. **b** the activity of ALP enzyme expressed as percentage of ALP activity of the magnetic cements compared to CPC, set as 100% (red dotted line). Bars indicate the mean values ± SD of results from three measurements in two separate experiments. Statistical analysis was performed using one-way analysis of variance (ANOVA), followed by Bonferroni’s multiple comparison test between samples. Statistically significant differences (*p* < 0.05) were observed between +/− SMF conditions within the same type of cement: *p* < 0.01 (**) and < 0.0001 (****). *p* < 0.0001 (****) were observed between MagCPC20 and MagOxCPC in SMF (+). No statistically significant differences (ns) between MagCPC20 and MagOxCPC in SMF (-)

The biological performance of the cement/scaffold in the presence (+) or absence (-) of static magnetic field (SMF) of 320–350 mT, was evaluated in terms of viability at different time points, morphology and cell distribution (Fig. 8). Furthermore, to evaluate the role of cements in cell differentiation, the activity of alkaline phosphatase was analysed (Fig. 9).

The viability of SaOs-2 cells was evaluated after 24 h, 72 h and 168 h, following their growth on cements MagCPC20 and MagOxCPC, in +/- SMF, compared to the CPC control cement (red dotted line, set at 100% of cell viability). The pH value of cell medium remained at the physiological value for the whole experiment.

Figure 8A demonstrates that SaOs-2 cells after 24 h, 72 h and 168 h on cements in SMF (−) show viability comparable to the CPC control and no statistically significant differences were observed between the two cements (*p* > 0.05). In contrast, the cells grown on MagCPC20 in SMF (+) condition (Fig. 8B) demonstrate higher viability, steadily enhanced up to 168 h.

Conversely, MagOxCPC induces a smaller increase in the cell viability in SMF (+) (with a clear decrease after 168 h), that can be correlated to the presence of magnetite, able to induce the onset of cell differentiation. Indeed, it has been already reported that SaOs-2 cell proliferation at a certain time declines when osteogenic markers such as ALP are expressed and become active, from this point on stimulating cell differentiation [32].

Furthermore, Fig. 8 illustrates the morphology (Fig. 8C, D) and the cells distribution (Fig. 8E, F) on MagCPC20 and MagOxCPC surfaces. A morphological analysis was conducted after 24 h to evaluate cell adhesion, using CLSM in SMF (−) (Fig. 8C) and SMF (+) (Fig. 8D). The CLSM images revealed that cells adhered to the surfaces after 24 h. This was evidenced by the formation of β-tubulin protein (in green), which is a component of the cytoskeleton, namely a network of protein filaments important to confer structural and mechanical properties to cells. The adherent cells in Fig. 8C, D seem in good condition in both +/− SMF, as evidenced by the nuclei (in blue), that in some cases are large, such as for the MagCPC20 in SMF (+) conditions (Fig. 8Da), indicating that cells are preparing to undergo mitosis. Moreover, Fig. 8E, F illustrate SEM images of well-distributed layer of cells after 7 days of growth on both cements in +/− SMF conditions. In SMF (−), cells formed a flat layer on MagCPC20 (Fig. 8Ea) and MagOxCPC (Fig. 8Eb). It should be noted that the MagOxCPC surface is not homogeneous, due to the discontinuity ascribable to the presence of magnetite as secondary phase, indeed lamellipodia can be observed attempting to establish connections between cells (Fig. 8Eb) [33, 34].

The application of SMF gives rise to extensive cell proliferation on the MagCPC20 surface (Fig. 8Fa). The SaOs-2 cells assume two distinct shapes: one type is spindle-like, well-spread on all the surface, while the other type is round and is present at the top, indicating extensive cell proliferation, as shown also in Fig. 8B. SEM images acquired on the MagOxCPC cement in SMF (+) (Fig. 8Fb) revealed the presence of a layer of cells distributed across multiple islands.

The observed differences in cell behaviour on the two different cements in SMF (+) (Fig. 8F) may be due to the different magnetization extent of the two cements (see Table 2). The MagCPC20 surface appears to enhance cell proliferation, as indicated by the quantitative data presented in Fig. 8B. Conversely, the MagOxCPC surface seems to decrease SaOs-2 cells growth (Fig. 8B), suggesting that cell differentiation has started at *t* = 168 h (Fig. 8Fb).

Images of cell morphology and distribution were acquired on CPC surfaces for comparative analysis. These images demonstrated that cells were adherent to the cements and in good condition after 24 h in both +/− SMF conditions (Supplementary Fig. S1, panels a, b). Following a 7-day growth period on CPC cement in +/− SMF conditions, cells formed a homogeneous layer (Supplementary Fig. S1, panels c, d).

Figure 9a reports the values of the extracted ALP protein: the obtained data demonstrate that SaOs-2 cells exhibit ALP expression slightly higher on MagCPC20 respect to MagOxCPC cements in SMF (−). However, when SMF is applied the difference in ALP expression becomes higher, due to the decreasing of the ALP expression on MagOxCPC. The quantified ALP on MagCPC20 in both +/− SMF conditions remains virtually unchanged.

It is conceivable that, despite the observed reduction in ALP expression on MagOxCPC in SMF (+) conditions, the enzyme may be more active. Indeed, as reported by [35], the ALP enzymatic activity may be high despite the low protein expression. Therefore, to evaluate cell differentiation, we studied the enzymatic activity as suggested by Feroz et al. [36] and our data illustrate that the presence of SMF enhanced cell differentiation on both magnetic cements and these findings are corroborated by viability results and SEM analyses (see Fig. 8).

Figure 9b reported the activity of the ALP enzyme with respect to the CPC control (red dotted line, set as 100% of ALP activity) in +/− SMF conditions after 7 days of cell incubation on the cements.

It is important to recall that the cements under investigation exhibit a similar concentration of calcium. The activity on the CPC surface is high as documented for the role of calcium ions in cellular processes including osteogenic differentiation [37]. It is noteworthy that in SMF (−) condition MagCPC20 exhibited ∼40% and MagOxCPC ∼ 55% of ALP activity, respectively, when compared to the CPC control. These results may be attributed to the lower calcium release from MagCPC20 compared to CPC and MagOxCPC (Fig. 7a). On the other hand, the presence of magnetite in MagOxCPC, leading to a concentration of iron twice than the one present in the MagCPC20 (see Table 3), may have inhibited ALP expression as suggested by Wang et al. [38], contrasting the positive effect given by its calcium content.

It is of interest to note that the application of SMF results in higher ALP activity, indicating that the presence of a 320–350 mT SMF has a significant effect in promoting cell differentiation on both the studied magnetic cements. This phenomenon has been observed in MagCPC20, which exhibited an increase of the ALP activity up to 60% and was particularly evident in MagOxCPC, which demonstrated a 110% activity. The data on higher ALP activity of magnetic cements in SMF (+) are in accordance with previous literature, i.e., Yang et al. [39] have reported that apatitic cements embedding iron oxides nanoparticles with a moderate SMF (1 mT - 1 T) promoted a higher osteoblast differentiation with respect to iron-free apatitic cement. No discernible change in cell distribution or ALP activity could be observed in CPC cement in SMF (+/−) (Supplementary Fig. S1 and Table S1).

It has been demonstrated in previous studies that iron, when taken up by cells, plays a crucial role in a multitude of cellular processes, including DNA synthesis [40] also favouring cell viability [41]. In the present work we observed that, besides the specific content in calcium and the extent of ions release, the ALP activity is influenced by the specific magnetisation of the cement where the cells are seeded. Indeed, the CPC cement exhibits no magnetization, whereas MagOxCPC shows higher magnetization than MagCPC20 (as shown in Table 2), and such a condition can have boosted the ALP activity in MagOxCPC.

## Discussion

In the present work, we developed and tested in vitro apatitic bone cements enriched with superparamagnetic nanoparticles as novel injectable bone scaffolds that can activate the regenerative process on demand, particularly relevant for the oral and maxillofacial district. In particular, the scope of this work is to demonstrate the biocompatibility and bioactivity of a new magnetic cement incorporating Fe^2+^/Fe^3+^-doped HA, to be compared with similar cements incorporating magnetite nanoparticles (SPIONs). Our results show that both magnetic cements (MagCPC20 and MagOxCPC) can be obtained by mixing CaP active precursor powder, superparamagnetic nanoparticles, and alginate with a liquid component, which yields injectable and easily workable pastes characterized by good cohesion, when extruded in saliva or other physiological fluids, thus generating mechanically consistent constructs. They show diffuse and open porosity promoting osteointegration, as evidenced by previous studies on the reference CPC cement [14, 42]. In addition, they are characterized by short setting times, reaching adequate mechanical performance suitable for their use in common surgical procedures. The two magnetic cements differ in respect to the form in which iron is present, which in turn influences the Fe ion release profile, the magnetization and, in consequence, the osteogenic profile: FeHA, embedded into the MagCPC20, is an apatite phase where Fe ions partially replace Ca^2+^ and are released under physiological conditions, while MagOxCPC embeds very stable iron oxide nanoparticles (magnetite), capable of conferring greater magnetisation but, on the other hand, negatively affecting long-term cytotoxicity. The release of iron from MagCPC20 is more than double in respect to the one of MagOxCPC, however the amount of iron ions released from MagCPC20 is very low, being less than 0.1 wt% (see Fig. 7). Considering the profile of the iron release curve, showing a plateau after 1 week, we suppose that FeHA maintains its magnetization for a long time, compliant with the bone remodelling, as we have confirmed by measuring the magnetization of the cement after 2 months of immersion in physiological fluid at 37 °C.

In vitro tests show that both magnetic cements slightly enhance cell proliferation, compared to the iron-free CPC cement. When static magnetic field is applied, cell proliferation is greatly enhanced with MagCPC20, whereas in the case of MagOxCPC only a slight boosting effect on cell proliferation after 72 h is detected, followed by a steep drop.

The ALP evaluation in terms of protein expression and enzymatic activity shows that both magnetic cements have high osteogenic ability, particularly the higher Ca ions release in MagOxCPC boosts the enzymatic activity compared to MagCPC20. On the other hand, the higher release of Fe ions in MagCPC20 negatively affects ALP enzymatic activity (in spite of the high expression of the protein), therefore, we can devise that the presence of Fe ions boosts the cell proliferation rather than cell differentiation. When SMF is applied, we observed that the ALP activity becomes higher in both cements, with a marked increase in MagOxCPC, and we ascribed this effect to its higher magnetization due to the presence of magnetite.

The functionalization with magnetic nanoparticles, either FeHA or magnetite (SPIONs), permitted the obtain cements endowed with superparamagnetic properties, thus capable of bio-activation on demand by the application of an external magnetic aligner. On the other hand, the assessed long-term cytotoxicity effect of SPIONs for their accumulation in the clearance organs [43] stimulated the research on alternative biomimetic phases endowed with superparamagnetic behaviour. For that reason, we studied Fe-doped HA as a biomimetic apatite phase, endowed with excellent biocompatibility and bioactivity as well as the ability to be magnetized under SMF [13, 44–47].

The use of magnetic fields is being increasingly explored for their ability to influence cell behaviour and regulate biochemical phenomena [48]. However, the investigation of the effects of specific magnetic fields on cell behaviour is still intriguing. Therefore, even though there is increasing evidence that magnetic fields, static, alternated, or pulsed, are relevant for enhancing tissue healing in the musculoskeletal system [49], much work is needed to ascertain the most appropriate magnetic fields to be applied, in terms of intensity and timing of application.

Targeting applications in dentistry, magnetic fields with designed intensity could be applied by using external devices such as aligners embedding small permanent magnets, that can be applied on demand [50, 51].

The present work puts in evidence the effectiveness of bone cements functionalized with magnetic apatites (FeHA), to enhance cell proliferation and osteogenesis under SMF. The magnetization extent of Fe-doped apatites can be adjusted during the synthesis process [15], thus representing a tuneable platform for the development of new therapies based on bioactive and completely safe magnetic cements. These materials can reduce foreign body reactions, thanks to their fully biomimetic composition, also preventing the use of other magnetic compounds such as SPIONs, potentially affecting the long term toxicity, as well as immune-inflammatory reactions [52–56]. In addition, the intrinsic antibacterial effect we already detected in similar apatitic bone cements [26] is predictable also for MagCPC20, and it can be further powered by the application of magnetic fields, as already observed in previous studies [57–59]. This is a relevant topic for further investigation in consideration of the high rate of post-operative infections occurring in dentistry [60–62]. Moreover, thanks to their ability to be powered by SMF easily applicable on demand, the use of magnetic apatitic cements in dentistry can be advantageous particularly for the treatment of patients with reduced endogenous regenerative potential due to ageing or to degenerative diseases such as periodontitis, whose number is steadily increasing and will count for the majority of patients in the incoming decades [63].

## Conclusions

Although the concept of magnetic activation in oral surgery is still in its very early stages, the potential benefits it offers are of clear impact. The use of synthetic implants in GBR procedures is increasingly relevant in dentistry, therefore, the possibility of providing precise control, non-invasiveness, and remote activation by external, non-destructive signals presents an exciting avenue for advancements in surgical procedures. The present work described a novel injectable cement containing FeHA nanopowder, designed to generate 3-D scaffold. Such devices can be activated on demand by the application of external magnetic signals, able to tune cell behaviour towards the osteogenic character. Further research and development in this area are needed to more precisely define the intensity and type of applied magnetic field, as well as the magnetization extent, which is influenced by the distance between the magnet and the bone defect.

The data obtained in this work are promising in paving the way for the adoption of easily injectable magnetic cements into routine practice in oral surgery. The application of user-friendly magnetic aligners able to activate the bone scaffold on demand, will improve the patient outcome, with particular interest for the elderly and patients with impaired conditions.

## Supplementary information

Supplementary Information

## Data Availability

The data supporting this article have been included as part of the Supplementary Information.

## References

[1] Khaohoen A, Sornsuwan T, Chaijareenont P, Poovarodom P, Rungsiyakull C, Rungsiyakull P. Biomaterials and clinical application of dental implants in relation to bone density—a narrative review. J Clin Med. 2023;12:6924–49. 37959389 10.3390/jcm12216924 PMC10649288

[2] Shakya A, Li Y, Chang Nwen, Liu X. Supra-alveolar bone regeneration: Progress, challenges, and future perspectives. Compos Part B Eng. 2024;283:111673. 10.1016/j.compositesb.2024.111673. 10.1016/j.compositesb.2024.111673 PMC11270636 39071449

[3] Terenzi V, Della Monaca M, Raponi I, Battisti A, Priore P, Barbera G, et al. MRONJ and ORNJ: When a single letter leads to substantial differences. Oral Oncol. 2020;110:104817. 10.1016/j.oraloncology.2020.104817 32475646

[4] Barbu HM, Iancu SA, Mirea IJ, Mignogna MD, Samet N, Calvo-Guirado JL. Management of schneiderian membrane perforations during sinus augmentation procedures: A preliminary comparison of two different approaches. J Clin Med. 2019;8:1491–505. 10.3390/jcm8091491 PMC6780245 31546766

[5] Elgali I, Omar O, Dahlin C, Thomsen P. Guided bone regeneration: materials and biological mechanisms revisited. Eur J Oral Sci. 2017;125:315–37. 28833567 10.1111/eos.12364 PMC5601292

[6] Tampieri A, Ruffini A, Ballardini A, Montesi M, Panseri S, Salamanna F, et al. Heterogeneous chemistry in the 3-D state: An original approach to generate bioactive, mechanically-competent bone scaffolds. Biomater Sci. 2019;7:307–21. 10.1039/c8bm01145a 30468436

[7] Sprio S, Fricia M, Maddalena GF, Nataloni A, Tampieri A. Osteointegration in cranial bone reconstruction: a goal to achieve. J Appl Biomater Funct Mater. 2016;14:470–6. 10.5301/jabfm.5000293. 10.5301/jabfm.5000293 27311430

[8] Sprio S, Preti L, Montesi M, Panseri S, Adamiano A, Vandini A, et al. Surface phenomena enhancing the antibacterial and osteogenic ability of nanocrystalline hydroxyapatite, activated by multiple-ion doping. ACS Biomater Sci Eng. 2019;5:5947–59. 33405685 10.1021/acsbiomaterials.9b00893

[9] Wu VM, Tang S, Uskoković V. Calcium phosphate nanoparticles as intrinsic inorganic antimicrobials: the antibacterial effect. ACS Appl Mater Interfaces. 2018;10:34013–28. 30226742 10.1021/acsami.8b12784

[10] Insua A, Galindo-Moreno P, Miron RJ, Wang H-L, Monje A. Emerging factors affecting peri‐implant bone metabolism. Periodontol 2000. 2024;94:27–78. 37904311 10.1111/prd.12532

[11] Santos LF, Silva AS, Mano JF. Magnetic-based strategies for regenerative medicine and tissue engineering. Adv Healthcare Mater. 2023;12:2300605. 10.1002/adhm.202300605 37543723

[12] Guan W, Gao H, Liu Y, Sun S, Li G. Application of magnetism in tissue regeneration: recent progress and future prospects. Regen Biomater. 2024;11:rbae048. 38939044 10.1093/rb/rbae048 PMC11208728

[13] Tampieri A, Iafisco M, Sandri M, Panseri S, Cunha C, Sprio S, et al. Magnetic bioinspired hybrid nanostructured collagen-hydroxyapatite scaffolds supporting cell proliferation and tuning regenerative process. ACS Appl Mater Interfaces. 2014;6:15697–707. 25188781 10.1021/am5050967

[14] Sprio S, Dapporto M, Montesi M, Panseri S, Lattanzi W, Pola E, et al. Novel Osteointegrative Sr-substituted apatitic cements enriched with alginate. Materials. 2016;9:763 28773884 10.3390/ma9090763 PMC5457115

[15] Tampieri A, D’Alessandro T, Sandri M, Sprio S, Landi E, Bertinetti L, et al. Intrinsic magnetism and hyperthermia in bioactive Fe-doped hydroxyapatite. Acta Biomater. 2012;8:843–51. 10.1016/j.actbio.2011.09.032. 22005331 10.1016/j.actbio.2011.09.032

[16] Iafisco M, Degli Esposti L, Ramírez-Rodríguez GB, Carella F, Gómez-Morales J, Ionescu AC, et al. Fluoride-doped amorphous calcium phosphate nanoparticles as a promising biomimetic material for dental remineralization. Sci Rep. 2018;8:1–9. 30451901 10.1038/s41598-018-35258-x PMC6242929

[17] Angelini E, Bianco P, Mascellani S, Zucchi F. Low-noble metal alloys: in vitro corrosion evaluation. J Mater Sci Mater Med. 1993;4:142–9.

[18] Duffó GS, Castillo Quezada. E. Development of an artificial saliva solution for studying the corrosion behavior of dental alloys. Corrosion. 2004;60:594–602.

[19] Pupilli F, Tavoni M, Drouet C, Tampieri A, Sprio S. Iron-doped hydroxyapatite by hydrothermal synthesis: Factors modulating the Fe2+, Fe3+ content. Open Ceram. 2024;18:100610. 10.1016/j.oceram.2024.100610.

[20] Bloise N, Patrucco A, Bruni G, Montagna G, Caringella R, Fassina L, et al. In vitro production of calcified bone matrix onto wool keratin scaffolds via osteogenic factors and electromagnetic stimulus. Materials. 2020;13:3052–73. 10.3390/ma13143052 PMC7411850 32650489

[21] Restivo E, Pugliese D, Gallichi-Nottiani D, Sammartino JC, Bloise N, Peluso E, et al. Effect of low copper doping on the optical, cytocompatible, antibacterial, and SARS-CoV-2 trapping properties of calcium phosphate glasses. ACS Omega. 2023;8:42264–74. 38024754 10.1021/acsomega.3c04293 PMC10652837

[22] Bloise N, Waldorff EI, Montagna G, Bruni G, Fassina L, Fang S, et al. Early Osteogenic Marker Expression in hMSCs cultured onto acid etching-derived micro-and nanotopography 3D-Printed Titanium surfaces. Int J Mol Sci. 2022;23:1–18. 10.3390/ijms23137070 PMC9266831 35806083

[23] Riva F, Bloise N, Omes C, Ceccarelli G, Fassina L, Nappi RE, et al. Human Ovarian follicular fluid mesenchymal stem cells express osteogenic markers when cultured on bioglass 58S-coated titanium scaffolds. Materials. 2023;16:1–16. 10.3390/ma16103676 PMC10222050 37241304

[24] Degli Esposti L, Adamiano A, Siliqi D, Giannini C, Iafisco M. The effect of chemical structure of carboxylate molecules on hydroxyapatite nanoparticles. A structural and morphological study. Bioact Mater. 2021;6:2360–71. 10.1016/j.bioactmat.2021.01.010. 33553821 10.1016/j.bioactmat.2021.01.010 PMC7844063

[25] Bohner M. Reactivity of calcium phosphate cements. J Mater Chem. 2007;17:3980–6.

[26] Dapporto M, Tavoni M, Restivo E, Carella F, Bruni G, Mercatali L, et al. Strontium-doped apatitic bone cements with tunable antibacterial and antibiofilm ability. Front Bioeng Biotechnol. 2022;10:1–17. https://www.frontiersin.org/articles/10.3389/fbioe.2022.969641/full. 10.3389/fbioe.2022.969641 PMC9780487 36568303

[27] Dricssens FCM, Flanell JA, Boltong MG, Khairoun I, Ginebra MP. Osteotransductive bone cements. Proc Inst Mech Eng Part H J Eng Med. 1998;212:427–35. 10.1243/0954411981534196 9852738

[28] Chen Z, Zhang W, Wang M, Backman LJ, Chen J. Effects of zinc, magnesium, and iron ions on bone tissue engineering. ACS Biomater Sci Eng. 2022;8:2321–35. 35638755 10.1021/acsbiomaterials.2c00368

[29] Pupilli F, Ruffini A, Dapporto M, Tavoni M, Tampieri A, Sprio S. Design strategies and biomimetic approaches for calcium phosphate scaffolds in bone tissue regeneration. Biomimetics. 2022;7:112. 35997432 10.3390/biomimetics7030112 PMC9397031

[30] Saino E, Maliardi V, Quartarone E, Fassina L, Benedetti L, De Angelis MGC, et al. In vitro enhancement of SAOS-2 cell calcified matrix deposition onto radio frequency magnetron sputtered bioglass-coated titanium scaffolds. Tissue Eng - Part A. 2010;16:995–1008. 19839719 10.1089/ten.TEA.2009.0051

[31] Prè D, Ceccarelli G, Benedetti L, Magenes G, De Angelis MGC. Effects of low-amplitude, high-frequency vibrations on proliferation and differentiation of SAOS-2 Human osteogenic cell line. Tissue Eng - Part C Methods. 2009;15:669–79. 19257810 10.1089/ten.TEC.2008.0599

[32] Owen TA, Aronow M, Shalhoub V, Barone LM, Wilming L, Tassinari MS, et al. Progressive development of the rat osteoblast phenotype in vitro: Reciprocal relationships in expression of genes associated with osteoblast proliferation and differentiation during formation of the bone extracellular matrix. J Cell Physiol. 1990;143:420–30. 1694181 10.1002/jcp.1041430304

[33] Rothamel D, Schwarz F, Sculean A, Herten M, Scherbaum W, Becker J. Biocompatibility of various collagen membranes in cultures of human PDL fibroblasts and human osteoblast-like cells. Clin Oral Implants Res. 2004;15:443–9. 15248879 10.1111/j.1600-0501.2004.01039.x

[34] You R, Li X, Liu Y, Liu G, Lu S, Li M. Response of filopodia and lamellipodia to surface topography on micropatterned silk fibroin films. J Biomed Mater Res - Part A 2014;102:4206–12. 10.1002/jbm.a.35097 24464986

[35] Nonoyama S, Karakida T, Chiba-Ohkuma R, Yamamoto R, Ujiie Y, Nagano T, et al. Development and characterization of alkaline phosphatase-positive human umbilical cord perivascular cells. Cells. 2021;10:3011–32. 10.3390/cells10113011 PMC8616437 34831233

[36] Feroz S, Muhammad N, Ullah R, Nishan U, Cathro P, Dias G. Mechanical properties, and in vitro biocompatibility assessment of biomimetic dual layered keratin/ hydroxyapatite scaffolds. Front Bioeng Biotechnol. 2023;11:1–14. 10.3389/fbioe.2023.1304147 PMC10764155 38173873

[37] Kito H, Ohya S. Role of K+ and Ca2+-permeable channels in osteoblast functions. Int J Mol Sci. 2021;22:10459–78. 10.3390/ijms221910459 PMC8509041 34638799

[38] Wang Y, Liang Z, Chen L, Yang G, Xu J, Deng C, et al. Protective effect of iron oxide nanoparticles on periodontal injury in rats by inhibiting Collagenase-1 and Alkaline Phosphatase Expression. J Biomed Nanotechnol. 2022;18:1131–7. 35854462 10.1166/jbn.2022.3322

[39] Yang J, Wu J, Guo Z, Zhang G, Zhang H. Iron oxide nanoparticles combined with static magnetic fields in bone remodeling. Cells. 2022;11:1–18. 10.3390/cells11203298 PMC9600888 36291164

[40] Peters K, Staehlke S, Rebl H, Jonitz-Heincke A, Hahn O. Impact of metal ions on cellular functions: a focus on mesenchymal stem/stromal cell differentiation. Int J Mol Sci. 2024;25:10127–53. 10.3390/ijms251810127 PMC11432215 39337612

[41] Adamiano A, Wu VM, Carella F, Lamura G, Canepa F, Tampieri A, et al. Magnetic calcium phosphates nanocomposites for the intracellular hyperthermia of cancers of bone and brain. Nanomedicine. 2019;14:1267–89. 10.2217/nnm-2018-0372 PMC6615412 31124760

[42] Montesi M, Panseri S, Dapporto M, Tampieri A, Sprio S. Sr-substituted bone cements direct mesenchymal stem cells, osteoblasts and osteoclasts fate. PLoS One. 2017;12:1–13. 10.1371/journal.pone.0172100 PMC5308610 28196118

[43] Dantas G de PF, Ferraz FS, Coimbra JLP, Paniago RM, et al. The toxicity of superparamagnetic iron oxide nanoparticles induced on the testicular cells: In vitro study. NanoImpact. 2024;35. 10.1016/j.impact.2024.100517 38848992

[44] Fernandes Patrício TM, Panseri S, Montesi M, Iafisco M, Sandri M, Tampieri A, et al. Superparamagnetic hybrid microspheres affecting osteoblasts behaviour. Mater Sci Eng C. 2019;96:234–47. 10.1016/j.msec.2018.11.014. 10.1016/j.msec.2018.11.014 30606529

[45] Panseri S, Cunha C, D’Alessandro T, Sandri M, Giavaresi G, Marcacci M, et al. Intrinsically superparamagnetic Fe-hydroxyapatite nanoparticles positively influence osteoblast-like cell behaviour. J Nanobiotechnol. 2012;10:1. 10.1186/1477-3155-10-32 PMC3458931 22828388

[46] Panseri S, Cunha C, D'Alessandro T, Sandri M, Russo A, Giavaresi G. Magnetic Hydroxyapatite bone substitutes to enhance tissue regeneration: Evaluation in vitro using osteoblast-like cells and in vivo in a bone defect. PLoS One. 2012;7:38710. 10.1371/journal.pone.0038710 PMC3369900 22685602

[47] Panseri S, Montesi M, Sandri M, Iafisco M, Adamiano A, Ghetti M, et al. Magnetic labelling of mesenchymal stem cells with iron-doped hydroxyapatite nanoparticles as tool for cell therapy. J Biomed Nanotechnol. 2016;12:909–21. 27305814 10.1166/jbn.2016.2248

[48] Hart DA. The influence of magnetic fields, including the planetary magnetic field, on complex life forms: how do biological systems function in this field and in electromagnetic fields? Biophysica. 2024;4:1–21.

[49] Iwasa K, Reddi AH. Pulsed electromagnetic fields and tissue engineering of the joints. Tissue Eng - Part B Rev. 2018;24:144–54. 29020880 10.1089/ten.teb.2017.0294 PMC5905856

[50] Shah A, Shah P, Goje SK, Shah R, Modi B. Use of magnetic forces in orthodontics: a review. Adv J Grad Res. 2017;1:30–4.

[51] Hoyberg C. United States Patent US9775687B1 - Magnetic tooth alignment devices and related methods. Filing date: 16 July 2015.

[52] Verma GS, Nirmal NK, Gunpal D, Gupta H, Yadav M, Kumar N, et al. Intraperitoneal exposure of iron oxide nanoparticles causes dose-dependent toxicity in Wistar rats. Toxicol Ind Health. 2021;37:763–75. 34797179 10.1177/07482337211058668

[53] Woo S, Kim S, Kim H, Cheon YW, Yoon S, Oh JH, et al. Charge-modulated synthesis of highly stable iron oxide nanoparticles for in vitro and in vivo toxicity evaluation. Nanomaterials. 2021;11:1–15. 10.3390/nano11113068 PMC8624538 34835832

[54] Yang CY, Hsiao JK, Tai MF, Chen ST, Cheng HY, Wang JL, et al. Direct labeling of hMSC with SPIO: The long-term influence on toxicity, chondrogenic differentiation capacity, and intracellular distribution. Mol Imaging Biol. 2011;13:443–51. 20567925 10.1007/s11307-010-0360-7

[55] Mahmoudi M, Hofmann H, Rothen-Rutishauser B, Petri-Fink A. Assessing the in vitro and in vivo toxicity of superparamagnetic iron oxide nanoparticles. Chem Rev. 2012;112:2323–38. 22216932 10.1021/cr2002596

[56] Mahmoudi M, Simchi A, Imani M, Shokrgozar MA, Milani AS, Häfeli UO, et al. A new approach for the in vitro identification of the cytotoxicity of superparamagnetic iron oxide nanoparticles. Colloids Surf B Biointerfaces. 2010;75:300–9. 19781921 10.1016/j.colsurfb.2009.08.044

[57] Bajpai I, Saha N, Basu B. Moderate intensity static magnetic field has bactericidal effect on E. coli and S. epidermidis on sintered hydroxyapatite. J Biomed Mater Res - Part B Appl Biomater 2012;100 B:1206–17. 10.1002/jbm.b.32685 22576793

[58] Brkovic S, Postic S, Ilic D. Influence of the magnetic field on microorganisms in the oral cavity. J Appl Oral Sci. 2015;23:179–86. 26018310 10.1590/1678-775720140243 PMC4428463

[59] Ciecholewska-Juśko D, Żywicka A, Junka A, Woroszyło M, Wardach M, Chodaczek G, et al. The effects of rotating magnetic field and antiseptic on in vitro pathogenic biofilm and its milieu. Sci Rep. 2022;12:1–19. 10.1038/s41598-022-12840-y. 35614186 10.1038/s41598-022-12840-y PMC9132948

[60] Yue Yi EK, Siew Ying AL, Mohan M, Menon RK. Prevalence of postoperative infection after tooth extraction: a retrospective study. Int J Dent. 2021;2021:6664311. 34211554 10.1155/2021/6664311 PMC8208874

[61] Dallaserra M, Poblete F, Vergara C, Cortés R, Araya I, Yanine N, et al. Infectious postoperative complications in oral surgery. An observational study. J Clin Exp Dent. 2020;12:e65–70. 31976046 10.4317/jced.55982 PMC6969960

[62] Camps-Font O, Figueiredo R, Valmaseda-Castellón E, Gay-Escoda C. Postoperative infections after dental implant placement: Prevalence, clinical features, and treatment. Implant Dent. 2015;24:713–9. 26384096 10.1097/ID.0000000000000325

[63] Suenaga H, Schifter M, Chen N, Ali F, Byth K, Peck C. Impact of oral/dental disease burden on postoperative infective complications: a prospective cohort study. Clin Oral Investig. 2023;27:6461–70. 10.1007/s00784-023-05251-4. 37730892 10.1007/s00784-023-05251-4 PMC10630249

