# Supplementary material for: Injectable bioactive scaffold able to stimulate oral bone regeneration on demand
Source: J Mater Sci Mater Med. 2025 Apr 8;36(1):31. doi: 10.1007/s10856-025-06879-2 (PMC11978537; doi:10.1007/s10856-025-06879-2)
Supplement: Supplementary file 1 — Supplementary Information [file 10856_2025_6879_MOESM1_ESM.docx]

SUPPLEMENTARY INFORMATION

Journal of Materials Science: Materials in Medicine

**Injectable bioactive scaffold able to stimulate oral bone regeneration on demand**

*Anna Tampieri^1*^, Marta Tavoni^1^, Teresa Vicidomini^2^, Hina Inam^1,3^, Elisa Restivo^4^, Livia Visai^4,5,6^, Umberto Romeo^2^, Simone Sprio^1*^*

^1^Institute of Science, Technology and Sustainability for Ceramics - Italian National Research Council (ISSMC-CNR), via Granarolo 64, Faenza 48018 Italy

^2^Department of Oral and Maxillofacial Sciences Sapienza University of Rome, Rome, Italy

^3^Department of Chemistry, Life Science and Environmental Sustainability, Parma University, Parco Area delle Scienze, 43124, Parma

^4^Molecular Medicine Department (DMM), Center for Health Technologies (CHT), UdR INSTM, University of Pavia, 27100 Pavia, Italy,

^5^UOR6 Nanotechnology Laboratory, Department of Prevention and Rehabilitation in Occupational Medicine and Specialty Medicine, Istituti Clinici Scientifici Maugeri IRCCS, Via Maugeri 4, 27100 Pavia, Italy

^6^Interuniversity Center for the Promotion of the 3Rs Principles in Teaching and Research (Centro 3R), Operative Unit (OU) of University of Pavia, 27100 Pavia, Italy

**E-mail addresses**

Anna Tampieri: [anna.tampieri@issmc.cnr.it](mailto:anna.tampieri@issmc.cnr.it)

Marta Tavoni: [marta.tavoni@issmc.cnr.it](mailto:marta.tavoni@issmc.cnr.it)

Teresa Vicidomini: [vicidomini.1859365@studenti.uniroma1.it](mailto:vicidomini.1859365@studenti.uniroma1.it)

Hina Inam: [hina.inam@issmc.cnr.it](mailto:hina.inam@issmc.cnr.it)

Elisa Restivo: [elisa.restivo01@universitadipavia.it](mailto:elisa.restivo01@universitadipavia.it)

Livia Visai: [livia.visai@unipv.it](mailto:livia.visai@unipv.it)

Umberto Romeo: [umberto.romeo@uniroma1.it](mailto:umberto.romeo@uniroma1.it)

Simone Sprio: [simone.sprio@issmc.cnr.it](mailto:simone.sprio@issmc.cnr.it)

***Corresponding Authors**

Anna Tampieri, ISSMC-CNR, Tel. +39 0546 699711, Fax: +39 0546 46381

Simone Sprio, ISSMC-CNR, Tel. +39 0546 699711, Fax: +39 0546 46381

**
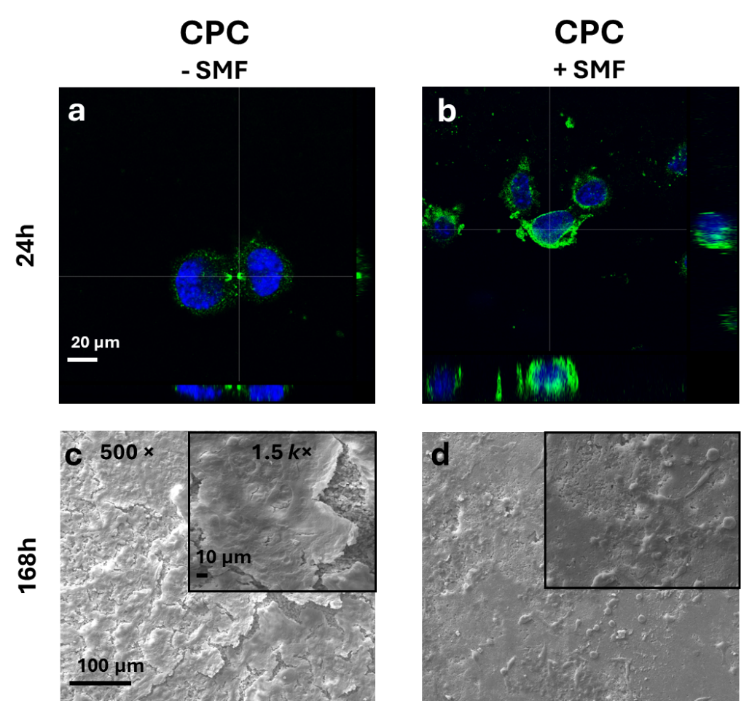
**

**Fig S1 –** Cell morphology and distribution. SaOs-2 cells were seeded on CPC control scaffold and incubated with proliferative medium for 24h, 72h and 168h, at 37°C + 5% CO_2_ in SMF (-) (a, c) and SMF (+) (b, d). Cell morphology was evaluated through CLSM (a-b) to investigate cell adhesion after 24h. SEM analysis (c-d) was performed after 168h to evaluate cell distribution on surfaces. a, b – CLSM images acquired with 2.5 electron zoom of 40× magnification (scale bar 20 µm). Cell nuclei were stained with Hoechst 33342 (in blue) and cytoskeletal β-tubulin with an anti-β tubulin antibody Alexa Fluor 488-conjugated (in green). c, d – SEM images of cells were acquired at 500× magnification (scale bar 100 µm) and insets at 1.5k× magnification (scale bar 10 µm). A well-formed flat layer of cells was observable on CPC scaffold in +/- SMF.

***
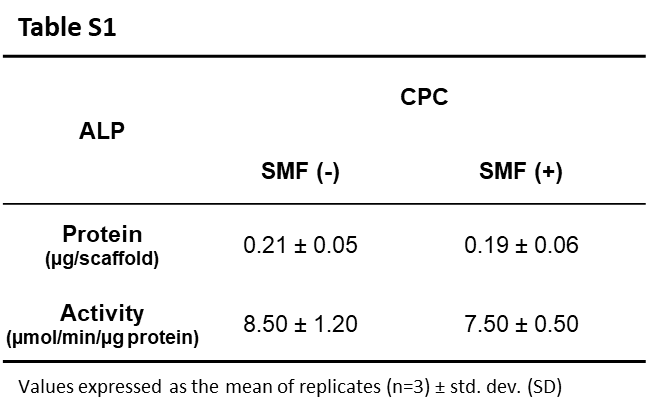
***

**Table S1** – Alkaline phosphatase quantification and activity on CPC. SaOs-2 cells were seeded on CPC scaffolds and incubated with proliferative medium for 7 days, at 37°C + 5% CO_2_. After evaluating cell viability, the proteins were extracted, and the quantity of alkaline phosphatase (ALP) was determined through ELISA assay and reported as micrograms produced on CPC scaffolds.

The activity of ALP enzyme was determined after 7 days of cell incubation. Data were corrected for micrograms of expressed ALP protein on CPC and reported as micromoles of *p*-nitrophenol produced per min per microgram of ALP protein. All data reported in the table were expressed as the mean values ± SD of results from three measurements in two separated experiments. No statistically significant differences (p > 0.05) were observed in ALP expression and ALP activity between +/- SMF conditions.
